# Hepatocellular Carcinoma Risk According to Regimens for Eradication of Hepatitis C Virus; Interferon or Direct Acting Antivirals

**DOI:** 10.3390/cancers12113414

**Published:** 2020-11-18

**Authors:** Hye Won Lee, Dai Hoon Han, Hye Jung Shin, Jae Seung Lee, Seung Up Kim, Jun Yong Park, Do Young Kim, Sang Hoon Ahn, Beom Kyung Kim

**Affiliations:** 1 Department of Internal Medicine, Yonsei University College of Medicine, Seoul 03722, Korea; lorry-lee@yuhs.ac (H.W.L.); sikarue@yuhs.ac (J.S.L.); ksukorea@yuhs.ac (S.U.K.); drpjy@yuhs.ac (J.Y.P.); dyk1025@yuhs.ac (D.Y.K.); ahnsh@yuhs.ac (S.H.A.); 2 Institute of Gastroenterology, Yonsei University College of Medicine, Seoul 03722, Korea; 3 Yonsei Liver Center, Severance Hospital, Seoul 03722, Korea; dhhan@yuhs.ac; 4 Liver Cancer Center, Yonsei Cancer Center, Yonsei University Health System, Seoul 03722, Korea; 5 Division of Hepatobiliary and Pancreatic Surgery, Department of Surgery, Yonsei University College of medicine, Seoul 03722, Korea; 6 Biostatistics Collaboration Unit, Department of Biomedical Systems Informatics, Yonsei University College of Medicine, Seoul 03722, Korea; hjshin105@yuhs.ac

**Keywords:** interferon, direct-acting antivirals, hepatocellular carcinoma, prognosis, comparison

## Abstract

**Simple Summary:**

Owing to pegylated interferon (PegIFN)-free direct-acting antivirals (DAAs) against chronic hepatitis C virus infection, a sustained virological response (SVR) rate >95% can be attained with a satisfactory tolerability and shorter treatment duration. However, it still remains controversial whether there is any difference in prognosis depending on treatment regimens—PegIFN or DAAs. After adjusting for imbalance between patients treated with PegIFN-based vs. DAA-based regimens, the post-SVR risk of hepatocellular carcinoma development was comparable according to treatment regimens. Furthermore, the risk was also similar between patients treated with sofosbuvir-based vs. sofosbuvir-free DAA regimens. Further studies with a longer follow-up period are required.

**Abstract:**

By pegylated interferon (PegIFN)-free direct-acting antivirals (DAAs) against hepatitis C virus (HCV) infection, a sustained virological response (SVR) rate >95% can be attained with a satisfactory tolerability and shorter treatment duration. However, it remains controversial whether there is any difference in prognosis depending on regimens—PegIFN or DAAs. We compared the probabilities of hepatocellular carcinoma (HCC) development between patients achieving an SVR by PegIFN/ribavirin (PegIFN group, n = 603) and DAAs (DAAs group, n = 479). The DAAs group was significantly older and had a higher proportion of cirrhosis than the PegIFN group. Before adjustment, the DAAs group had a higher HCC incidence than the PegIFN group (*p* < 0.001). However, by multivariate analyses, the DAAs (vs. PegIFN) group was not associated with HCC risk (adjusted hazard ratio 0.968, 95% confidence interval 0.380–2.468; *p* = 0.946). Old age, male, higher body mass index, cirrhosis, and lower platelet count were associated with increased HCC risk (all *p* < 0.05). After propensity score matching (PSM), a similar HCC risk between the two groups was observed (*p* = 0.372). We also compared HCC incidences according to sofosbuvir (SOF)-based and SOF-free DAAs, showing a similar risk in both groups before adjustment (*p* = 0.478) and after PSM (*p* = 0.855). In conclusion, post-SVR HCC risks were comparable according to treatment regimens; PegIFN- vs. DAA-based regimens and SOF-based vs. SOF-free DAA regimens. Further studies with a longer follow-up period are required.

## 1. Introduction

Hepatitis C virus (HCV) is globally one of the major health problems causing hepatocellular carcinoma (HCC) and/or cirrhosis [1]. Successful eradication of chronic HCV infection through antiviral treatment has proved to reduce the risk of such liver disease progression. Previously, antiviral treatments centered on the use of interferon-alpha (IFN)-based regimens. However, pegylated IFN (PegIFN)/ribavirin (RBV) not only has various kinds of adverse effects with a long dose administration period, i.e., at least 24 to 48 weeks, but also an inevitably high rate of treatment discontinuation. Therefore, its wide use is substantially limited, especially for patients with old age, decompensated cirrhosis, or other comorbidities. In addition, more importantly, their sustained virological response (SVR) rates are also sub-optimal. Currently, primarily owing to the introduction of PegIFN-free direct-acting antivirals (DAAs) in 2014, an SVR rate >95% can be attained with satisfactory tolerability [2,3,4,5,6,7].

Meanwhile, the risk of HCC development has not been well-controlled in real-life practice, even after achievement of SVR. In the early period since PegIFN-free DAA regimens became available, data showed the paradoxically tumorigenic potential, facilitating the early occurrence and recurrence of HCC following a DAA-induced SVR [8,9]. Furthermore, in an article about cost-effective analysis of post-SVR HCC surveillance, HCC incidences after DAA-induced SVR were adopted at 0.34~1.82%/years, slightly higher than those after IFN-induced SVR of 0.23~1.39%/years [10]. Several molecular studies theoretically supported such a phenomenon by indicating the potential effect of DAAs on angiogenesis and cellular proliferation [11,12,13]. In a similar context, another study reported that sofosbuvir (SOF) may activate epidermal growth factor receptor (EGFR)-dependent pathways, inducing liver-related pathological processes [14]. Nevertheless, subsequent studies have failed to reproduce such kinds of data systematically yet, indicating that there has been no difference in the risk of HCC occurrence or recurrence following SVR from DAA- vs. IFN-based therapy so far [15,16,17,18]. However, Ogawa et al. [19] recently proposed a trend toward a higher short-term HCC incidence in a subgroup treated with a specific DAA, i.e., SOF.

In this study, we aimed to compare the preventive effect of HCV eradication by PegIFN/RBV vs. DAAs on liver disease progression, including the risk of HCC development, and to assess differences in HCC incidence according to the type of DAA.

## 2. Methods

### 2.1. Patients

From 2011 to 2019, treatment-naïve patients with chronic HCV infection who were treated with PegIFN /RBV regimen or DAAs (referred as “PegIFN group” and “DAAs group”, respectively) at the Severance Hospital, Yonsei University College of Medicine, Seoul, Republic of Korea, were screened for eligibility. The inclusion criteria were as follows: (1) age ≥ 19 years; (2) detectable serum HCV-RNA before antiviral treatment; and (3) achievement of SVR after antiviral treatment. Exclusion criteria were as follows: (1) simultaneous administration of PegIFN plus DAAs; (2) previous antiviral treatment against HCV infection; (3) history of HCC or hepatic decompensation; (4) co-infection with other hepatitis viruses; (5) reinfection after SVR; (6) history of organ transplantation; (7) HCC development, hepatic decompensation, or death within 6 months of enrollment; and (8) other significant medical illnesses. PegIFN/RBV or DAA regimens were prescribed according to the practice guideline [20,21] and SVR was defined as undetectable serum HCV-RNA with a lower limit of quantification of 15 IU/mL at 24 or 12 weeks after PegIFN/RBV or DAA regimens, respectively. If histologic information was not available, cirrhosis was clinically defined as follows: (1) platelet count <150,000/μL and ultrasonographic findings suggestive of cirrhosis, including a blunted, nodular liver edge accompanied by splenomegaly (>12 cm); or (2) esophageal or gastric varices [22].

The study was approved by the institutional review board of the Severance Hospital and conformed to the ethical guidelines of the 1975 Helsinki Declaration (IRB No. 4-2017-1217).

### 2.2. Clinical Evaluation and Study Endpoints

After achievement of SVR, all patients received laboratory tests every 6 months and underwent periodic surveillance with ultrasonography and serum alpha-fetoprotein levels to screen for HCC and cirrhotic complications every 6 months [23,24,25].

The primary outcome was the cumulative risk of HCC development. HCC was diagnosed based on histological evidence or radiological findings determined by dynamic computed tomography and/or magnetic resonance imaging (nodule > 1 cm with arterial hyper-vascularity and portal/delayed-phase washout) [26,27,28,29]. In addition, we also investigated the cumulative risk of cirrhotic complication events (CCEs), including shift to Child–Pugh class B, presence of related symptoms (e.g., ascites, variceal hemorrhage, hepatorenal syndrome, or hepatic encephalopathy), cirrhosis-related death, or liver transplantation. To avoid statistical repetition in a patient experiencing different types of cirrhotic complications at different times, we selected the first event of cirrhotic complications for statistical analysis.

### 2.3. Statistical Analysis

Data are expressed as means ± standard deviations, medians (IQRs), or no. (%), as appropriate. Differences among continuous and categorical variables were examined for statistical significance by the Student’s *t*-test (or the Mann–Whitney test, if appropriate) and chi-squared (or Fisher’s exact tests, if appropriate) test.

The cumulative risk of development of HCC or CCEs was evaluated using the Kaplan–Meier method, with comparisons using the log-rank test. As DAAs in the Republic of Korea became widely prescribed since 2015, the follow-up was censored at 48 months. In order to assess the associations between the risk of HCC or CCEs and each variable and to calculate hazard ratios (HRs) and 95% confidence intervals (CIs), a Cox regression analysis was performed. Then, a multivariable Cox regression analysis was performed to determine the final prognostic factors associated with disease progression. Furthermore, to reduce the effect of selection bias and potential confounders between the PegIFN and DAA groups, propensity score (PS) was calculated using logistic regression. Then, PS matching analyses were performed to compare the cumulative risks of HCC or CCEs between the two groups.

All statistical analyses were conducted using SAS, version 9.2 (SAS Institute), R (V.3.0, http://cran.r-project.org/), and IBM^®^ SPSS^®^ Statistics for Windows, version 25.0 (IBM Corp., Armonk, NY, USA). Two-sided *p*-values < 0.05 were considered to indicate statistical significance.

## 3. Results

### 3.1. Patients’ Characteristics

According to the recruitment criteria, a total of 1082 patients were analyzed (Figure S1). Patients’ characteristics at the time of SVR are described in Table 1. The DAAs group (n = 603) members were significantly older (mean 61.0 vs. 49.5 years, respectively; *p* < 0.001) and had a lower proportion of males (38.0 vs. 44.7%, respectively; *p* = 0.029), lower aspartate aminotransferase (AST) (mean 25.0 vs. 32.5 IU/mL, respectively; *p* < 0.001), alanine aminotransferase (ALT) (mean 19.7 vs. 29.1 IU/mL, respectively; *p* < 0.001), and albumin (mean 4.1 vs. 4.3 g/dL, respectively; *p* < 0.001) levels, and higher total bilirubin (mean 0.9 vs. 0.7 mg/dL, respectively; *p* < 0.001), platelet count levels (mean 186 vs. 161 × 10^3^/uL, respectively; *p* < 0.001), and alpha-fetoprotein (AFP) (median 3.26 vs. 2.45 ng/mL, respectively), in comparison with the PegIFN group (n = 479). The proportion of HCV genotype 2 was significantly higher in the PegIFN group than in the DAAs group (60.8 vs. 46.3%, respectively; *p* < 0.001). The most commonly used first-line DAAs regimen was daclatasvir/asunaprevir (36.8%), followed by SOF/RBV (34.2%) (Table 2). Among the entire population, the most common HCV genotype was 2 (n = 570), followed by genotyped 1 (n = 496), 3 (n = 14), 4 (n = 1), and 6 (n = 1).

### 3.2. Changes in the Fibrotic Burden before and after Treatments

We assessed the fibrotic burden before and after treatment, using the FIB-4 index [30]. Before treatment, the DAAs group had a significantly higher FIB-4 index than the PegIFN group (2.62 [IQR 1.47~5.33] vs. 1.67 [IQR 0.97~3.04], respectively; *p* < 0.001). However, the DAAs group showed a significantly favorable difference rate of FIB-4 index from the pre-treatment value than the PegIFN group (23.9% [IQR 5.3~44.7] vs. −11.0% [IQR −52.6~23.9], respectively; *p* < 0.001). Consequently, there was no significant difference in the post-SVR FIB-4 index between the DAAs and PegIFN groups—1.86 (IQR 1.18~3.09) vs. 1.73 (IQR 1.11~2.91), respectively (*p* = 0.142).

### 3.3. Predictive Factors for HCC and CCE Development

**Figure 1:**
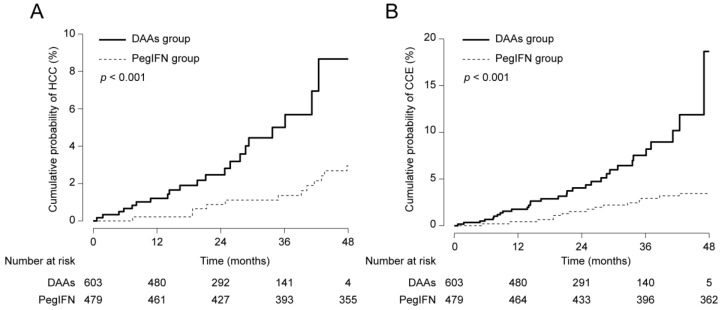
Cumulative probabilities of hepatocellular carcinoma (HCC) (**A**) and cirrhotic complication event (CCE) (**B**) development between the DAAs and PegIFN groups before statistical adjustment.

During the follow-up, a total of 33 (3.0%) and 47 (4.3%) patients developed HCC and CCE, respectively. Before statistical adjustment, the cumulative probabilities of both HCC (Figure 1A) and CCE (Figure 1B) development were significantly higher in the DAAs group than in the PegIFN group (both *p* < 0.001). To adjust bias between two groups, multivariate analyses were performed on the basis of significant univariate predictors with *p* < 0.05 (Table 3). In terms of the risk of HCC development, antiviral regimens (DAAs vs. PegIFN groups) did not affect the final prognosis, with adjusted HR (aHR) of 0.968 (95% CI 0.380~2.468; *p* = 0.946). Instead, old age (aHR 1.104, 95% CI 1.055~1.155; *p* < 0.001), male (aHR 2.990, 95% CI 1.365~6.550; *p* = 0.006), body mass index (aHR 1.127, 95% CI 1.023~1.241, *p* = 0.015), liver cirrhosis (aHR 4.861, 95% CI 1.706~13.849; *p* = 0.003) and lower platelet count (aHR 0.988, 95% CI 0.977~0.998; *p* = 0.023) at the time of SVR were independently associated with an increased risk of HCC development.

The contents of CCEs were as follows; varix bleeding (n = 11), ascites (n = 32), hepatic encephalopathy (n = 2), hepatorenal syndrome (n = 1), or liver transplantation (n = 1). Likewise, in terms of the risk of CCE development, antiviral regimens (DAAs vs. PegIFN groups) did not affect the final prognosis, with an aHR of 1.840 (95% CI 0.858~3.945; *p* = 0.117). Instead, old age (aHR 1.035, 95% CI 1.003~1.067; *p* = 0.032), diabetes (aHR 1.815, 95% CI 1.013~3.251; *p* = 0.045), and liver cirrhosis (aHR 2.267, 95% CI 1.095~4.696; *p* = 0.028) at the time of SVR were independently associated with an increased risk of CCE development.

### 3.4. The Risk of HCC and CCE Development after PS Matching Analysis

**Figure 2:**
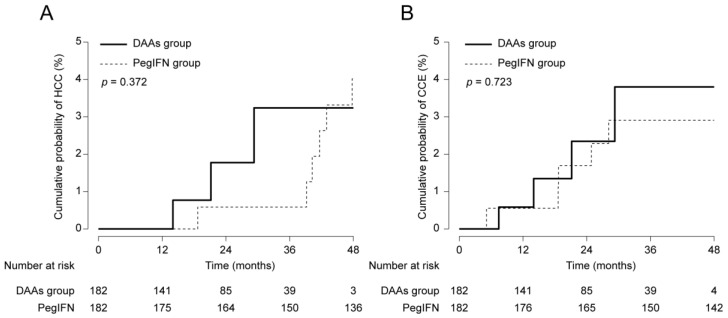
Cumulative probabilities of HCC (**A**) and CCE (**B**) development between the DAAs and PegIFN groups after propensity score (PS) matching analysis.

PS was calculated based upon variables at the time of SVR; age, male gender, diabetes, ALT, albumin, total bilirubin, platelet count, and liver cirrhosis. PS matching with a 1:1 ratio generated 182 pairs, where variables were well-balanced (all *p* > 0.05). Like the results shown from multivariate analyses, similar phenomena were also observed; there was no significant difference in terms of the risk of HCC (Figure 2A; *p* = 0.372) and CCE (Figure 2B; *p* = 0.723) development.

### 3.5. Subgroup Analyses among the DAAs Group

**Figure 3:**
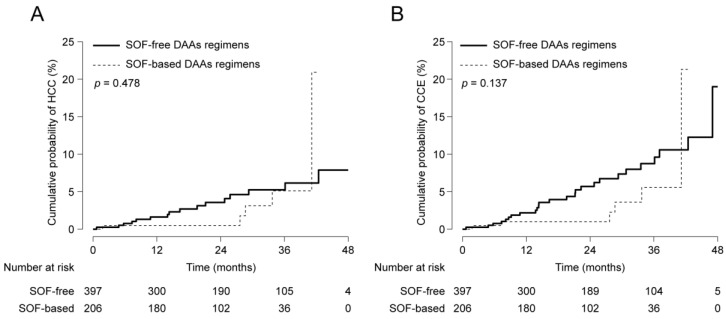
Cumulative probabilities of HCC (**A**) and CCE (**B**) development between patients treated with the SOF-based and the SOF-free DAAs regimens before statistical adjustment.

**Figure 4:**
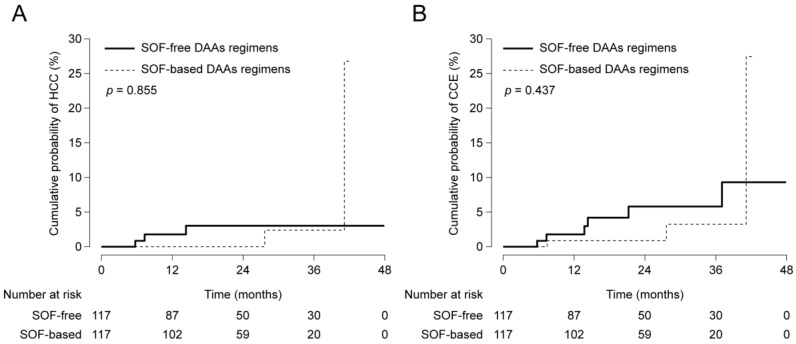
Cumulative probabilities of HCC (**A**) and CCE (**B**) development between patients treated with SOF-based and SOF-free DAAs regimens after PS matching analysis.

Among the DAAs group, we also compared the risk of HCC and CCE development according to the specific DAA regimen, i.e., SOF. The clinical characteristics of patients treated with the SOF-based DAAs regimen (n = 206) and with the SOF-free DAAs regimen (n = 397) are described in Table 4. Before adjustment, there was no significant difference between the two groups in terms of the risk of HCC (Figure 3A; *p* = 0.478) and CCE (Figure 3B; *p* = 0.137) development. To adjust bias between the two groups, PS was calculated using the above-mentioned method. PS matching with a 1:1 ratio generated 117 pairs, where variables were well-balanced (all *p* > 0.05). Similar results were also reproduced; there was no significant difference in terms of the risk of HCC (Figure 4A; *p* = 0.855) and CCE (Figure 4B; *p* = 0.437) development.

### 3.6. Risk Stratification According to Post-SVR FIB-4 Index

In order to identify a subgroup where the universal biannual HCC surveillance program might be safely withdrawn or its interval might be prolonged, we stratified the cumulative risk of HCC development according to a post-SVR FIB-4 index on the basis of another study [10]. Patients with post-SVR FIB-4 index <2.75 (n = 773, 71.4%) and <3.75 (n = 890, 82.3%) have annual HCC incidences of <0.2%/years and <0.5%/years, respectively, whereas those with post-SVR FIB-4 index ≥2.75 (n = 309, 28.6%) and ≥3.75 (n = 192, 17.7%) have annual HCC incidences of 3.5%/years and 4.0%/years, respectively (Figures S2 and S3, respectively).

The areas under the receiver operating characteristic curve were 0.837 (95% CI 0.780–0.895) for HCC development and 0.772 (95% CI 0.704–0.840) for CCE development.

## 4. Discussion

In this study, there was no significant difference in the risk of liver disease progression regarding HCC and CCE development between two groups following statistical adjustment, even though the DAAs group had higher incidences of both HCC and CCE developments than the PegIFN group before adjustment. Notably, before adjustment, the DAAs group had significantly unfavorable pre-treatment parameters, such as older age and a higher proportion of liver cirrhosis, compared to the PegIFN group. In addition, there was no significant difference in clinical prognosis between those undergoing SOF-based and SOF-free DAAs regimens in the present study, despite the in vitro analyses showing that SOF, in comparison with other DAAs, i.e., simeprevir and daclatasvir, tends to activate EGFR-dependent signaling pathways facilitating hepato-carcinogenesis [14]. As the systemic exposure to SOF during antiviral treatment is limited, usually within 12 weeks, its theoretically unfavorable effect may not directly translate into the worse clinical outcomes. However, Rinaldi et al. [31] recently indicated that patients undergoing SOF-based regimens without ribavirin have a 5.7 times higher risk of HCC than those undergoing RBV-based regimens with or without SOF. Hence, future studies regarding the risk of HCC according to the specific type of DAAs regimens will be needed to address such controversies.

Our study has several strengths. First, all of the patients enrolled in our study, including those without underlying advanced fibrosis or cirrhosis, underwent a routine biannual HCC surveillance based upon abdominal ultrasonography and serum alpha-fetoprotein during antiviral treatment and the follow-up after SVR. Therefore, we could establish the appropriate timing of HCC diagnosis, and the HCC incidence observed in our study became much more reliable and practical through prevention of under-estimation [32]. Second, a large sample size (>1000), an appropriate number of clinical events of 3~4%, given the follow-up duration with a medium term, and the consistently re-produced results after adjustment can provide statistically robust evidence. In a similar context, detailed laboratory data available from a hospital-oriented cohort and a good adherence shown during the follow-up allowed us to evaluate any differences in the HCC incidence more reliably between groups. Last, in order to identify a subgroup where the universal HCC surveillance program might be safely withdrawn or its interval might be prolonged, for example, from bi-annually to annually, we suggested the criteria of post-SVR FIB-4 index of <2.75 according to an annual HCC incidence rate of <0.2%. By this criteria, approximately 71.4% of the study population can avoid unnecessary visit to the clinic. To the best of our knowledge, studies assessing HCC risk on the basis of post-SVR FIB-4 index instead of pre-treatment FIB-4 index are relatively scarce so far. Current practice guidelines usually recommend a post-SVR HCC surveillance of high-risk patients, i.e., patients with underlying advanced fibrosis or cirrhosis [33]. However, whether such kinds of HCC screening are necessary among so called “low-risk” patients still remains controversial. For example, the European Association for the Study of the Liver (EASL) recommends that patients with SVR should be discharged unless they have advanced fibrosis/cirrhosis or other risk factors [34]. However, HCC still may occur in so-called low-risk patients after HCV eradication and its risk might persist for >10 years after SVR [35]. So, the Asian Pacific Association for the Study of the Liver (APASL) recommends that patients with SVR should be followed up at different intervals based on their underlying risks [36]. Further large-scale studies are required to address this issue in terms of not only the medical but also socio-economical perspectives.

The DAAs group had a higher pre-treatment fibrotic burden, estimated through the FIB-4 index, than the PegIFN group. Considering the various disadvantages of PegIFN based regimens, such phenomena might be generally plausible in the practical milieu. The significantly higher proportion of HCV genotype 2 among the PegIFN group also supported such a trend given the higher SVR rate with the shorter treatment duration for patients with HCV genotype 2. Nevertheless, it is noteworthy that the post-SVR fibrotic burdens between two groups were comparable. Accordingly, the DAAs group showed better anti-fibrotic efficacy expressed as a higher rate of decrease in FIB-4 index than the PegIFN group.

There are several unresolved issues in the present study. First, since this study was conducted using a single tertiary referral hospital-based cohort, the findings are potentially subject to selection bias. However, a homogeneous study population, a statistically reliable sample size and event number, and consistent results through various kinds of statistical adjustment could help to overcome this drawback. Second, in the Republic of Korea, most patients are infected with HCV genotypes 1b or 2 [37]. Thus, these results may not be generalizable for the full spectrum of the population with chronic HCV infection, from genotypes 1 to 6. Therefore, further validation studies are required. Third, given the late timing of reimbursement of DAA regimens in the Republic of Korea, studies with a long-term follow-up duration of >5 years are required in the near future in order to assess the risk of HCC development more accurately. In a similar context, to draw a robust conclusion regarding the preventive effect according to the DAA types, further studies including patients treated with newer DAA regimens, i.e., sofosbuvir/velpatasvir or sofosbuvir and velpatasvir/voxilaprevir, are also necessary [34].

## 5. Conclusions

In conclusion, among patients achieving SVR, the overall clinical prognosis was comparable according to antiviral regimens; PegIFN- vs. DAA-based regimens among the entire population and SOF-based vs. SOF-free regimens among the DAAs group. Further studies with a longer follow-up period should be conducted.

## Figures and Tables

**Table 1:** Comparison of clinical characteristics at the time of sustained virological response (SVR) between two groups (n = 1082).

Variables	Total (n = 1082)	DAAs Group (n = 603)	PegIFN Group (n = 479)	*p* Value
Age, years	55.9 ± 13.7	61.0 ± 12.8	49.5 ± 12.0	<0.001
Male gender, no. (%)	443 (40.9)	229 (38.0)	214 (44.7)	0.029
Body mass index, kg/m^2^	23.8 ± 3.3	23.8 ± 3.4	23.7 ± 3.2	0.831
Diabetes, no. (%)	240 (22.2)	145 (24.0)	95 (19.8)	0.056
AST, IU/mL	28.3 ± 21.8	25.0 ± 13.5	32.5 ± 28.5	<0.001
ALT, IU/mL	23.9 ± 22.3	19.7 ± 14.9	29.1 ± 28.1	<0.001
Total bilirubin, mg/dL	0.8 ± 0.4	0.9 ± 0.5	0.7 ± 0.3	<0.001
Albumin, g/dL	4.2 ± 0.4	4.1 ± 0.4	4.3 ± 0.4	<0.001
Platelet count, ×10^3^/uL	175 ± 70	186 ± 72	161 ± 64	<0.001
Creatinine, mg/dL	0.9 ± 1.1	0.9 ± 1.0	0.9 ± 1.1	0.870
AFP, ng/mL	2.91 (2.05~4.35)	3.26 (2.35~4.86)	2.45 (1.82~3.55)	<0.001
FIB-4 index	1.80 (1.16~3.00)	1.86 (1.18~3.09)	1.73 (1.11~2.91)	0.142
Liver cirrhosis, no. (%)	247 (22.8)	165 (27.4)	82 (17.1)	<0.001
HCV genotype 2, no. (%)	570 (52.7)	279 (46.3)	291 (60.8)	<0.001

Abbreviations: SVR, sustained virological response; DAAs, direct-acting antivirals; PegIFN, pegylated interferon; AST, aspartate aminotransferase; ALT, alanine aminotransferase; AFP, alpha-fetoprotein; HCV, hepatitis C virus.

**Table 2:** Types of DAAs (n = 603).

DAAs	No. (%)
Daclatasvir + asunaprevir	222 (36.8)
SOF + RBV	206 (34.2)
Glecaprevir + pibrentasvir	49 (8.1)
Elbasvir + grazoprevir	44 (7.3)
Ledipasvir + SOF	41 (6.8)
Ombitasvir + paritaprevir + ritonavir + dasabuvir	41 (6.8)

Abbreviations: DAAs, direct-acting antivirals; SOF, sofosbuvir; RBV, ribavirin.

**Table 3:** Predictive factors for clinical outcomes (n = 1082).

	HCC Development				CCE Development			
	Univariate	Multivariate			Univariate	Multivariate		
Variables	*p* Values	Adjusted HR	95% CI	*p* Values	*p* Values	Adjusted HR	95% CI	*p* Values
Age	<0.001	1.104	1.055–1.155	<0.001	<0.001	1.035	1.003–1.067	0.032
Male gender	0.006	2.990	1.365–6.550	0.006	0.063	-	-	-
BMI, kg/m^2^	0.004	1.127	1.023–1.241	0.015	0.127	-	-	-
Diabetes	<0.001	2.002	0.946–4.238	0.070	0.001	1.815	1.013–3.251	0.045
Liver cirrhosis	<0.001	4.861	1.706–13.849	0.003	<0.001	2.267	1.095–4.696	0.028
FIB-4 index	<0.001	0.949	0.827–1.090	0.460	<0.001	1.002	0.906–1.108	0.971
Total bilirubin	0.009	0.823	0.316–2.145	0.690	<0.001	1.298	0.672–2.505	0.438
Albumin	<0.001	0.783	0.321–1.912	0.592	<0.001	0.603	0.291–1.249	0.174
Platelet count	<0.001	0.988	0.977–0.998	0.023	<0.001	0.996	0.989–1.003	0.259
AFP, ng/mL	0.566	-	-	-	0.641	-	-	-
DAAs (vs. PegIFN) group	0.001	0.968	0.380–2.468	0.946	<0.001	1.840	0.858–3.945	0.117

Abbreviations: HCC, hepatocellular carcinoma; CCE, cirrhotic complication event; HR, hazard ratio; 95% CI, 95% confidence interval; BMI, body mass index; FIB-4, fibrosis-4; AFP, alpha-fetoprotein; DAAs, direct-acting antivirals; PegIFN, pegylated interferon.

**Table 4:** Comparison of clinical characteristics between patients treated with SOF-based and SOF-free DAA regimens.

Variables	SOF-Based DAA Regimens (n = 206)	SOF-Free DAA Regimens (n = 397)	*p* Value
Age, years	61.3 ± 12.2	60.9 ± 13.1	0.675
Male gender, no. (%)	76 (36.9)	153 (38.5)	0.380
Body mass index, kg/m^2^	23.7 ± 3.2	23.8 ± 3.4	0.747
Diabetes, no. (%)	46 (22.3)	99 (24.9)	0.272
AST, IU/mL	24.5 ± 14.7	25.3 ± 12.9	0.502
ALT, IU/mL	17.9 ± 12.3	20.6 ± 16.1	0.034
Total bilirubin, mg/dL	1.0 ± 0.5	0.8 ± 0.4	<0.001
Albumin, mg/dL	4.1 ± 0.4	4.2 ± 0.4	0.108
Platelet count, ×10^3^/uL	201 ± 76	178 ± 68	<0.001
Creatinine, mg/dL	0.8 ± 0.4	1.0 ± 1.2	<0.001
AFP, ng/mL	3.28 (2.38–5.16)	3.24 (2.34–4.83)	0.880
FIB-4 index	1.59 (1.15–2.77)	1.88 (1.21–2.88)	0.142
Liver cirrhosis, n (%)	55 (26.7)	110 (27.7)	0.435
Values were assessed at the time of sustained virologic response and expressed as mean ± standard deviation or no. (%).			

Abbreviations: SOF, sofosbuvir; DAAs, direct-acting antivirals; AST, aspartate aminotransferase; ALT, alanine aminotransferase; INR, international normalized ratio.

## Supplementary Materials

The following are available online at https://www.mdpi.com/2072-6694/12/11/3414/s1, Figure S1: Flow chart of study population, Figure S2: Cumulative probabilities of HCC between patients with FIB-4 ≥ 2.75 and those with FIB-4 < 2.75, Figure S3: Cumulative probabilities of HCC between patients with FIB-4 ≥ 3.75 and those with FIB-4 < 3.75.
