# Supplementary material for: Hepatocellular Carcinoma Risk According to Regimens for Eradication of Hepatitis C Virus; Interferon or Direct Acting Antivirals
Source: Cancers (Basel). 2020 Nov 18;12(11):3414. doi: 10.3390/cancers12113414 (PMC7698608; doi:10.3390/cancers12113414)
Supplement: Supplementary file 1 [file cancers-12-03414-s001.pdf]

Supplementary Materials:

Hepatocellular Carcinoma Risk According to Regimens for Eradication of Hepatitis C Virus; Interferon or Direct Acting Antivirals

Hye Won Lee [1,2,3,4,†], Dai Hoon Han [3,4,5,†], Hye Jung Shin [6], Jae Seung Lee [1,2,3,4], Seung Up Kim [1,2,3,4], Jun Yong Park [1,2,3,4], Do Young Kim [1,2,3,4], Sang Hoon Ahn [1,2,3,4] and Beom Kyung Kim [1,2,3,4,*]

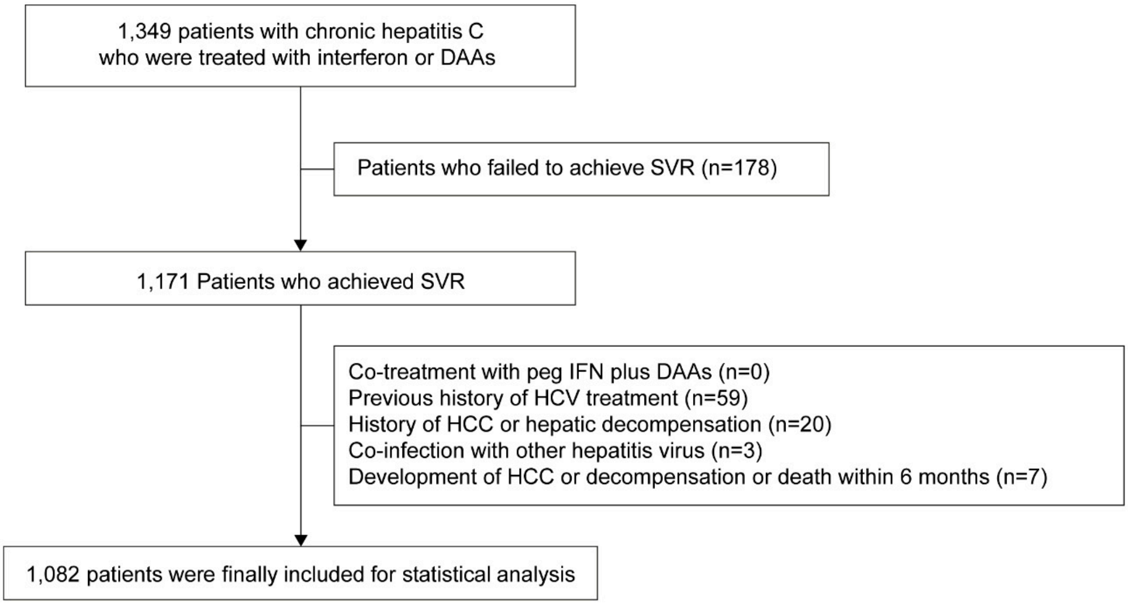

Figure S1. Flow chart of study population.

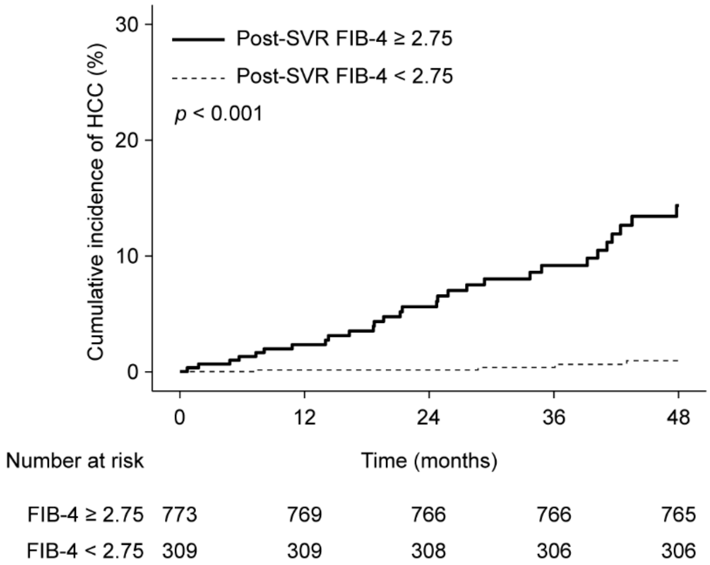

Figure S2. Cumulative probabilities of HCC between patients with FIB-4 ≥ 2.75 and those with FIB-4 < 2.75.

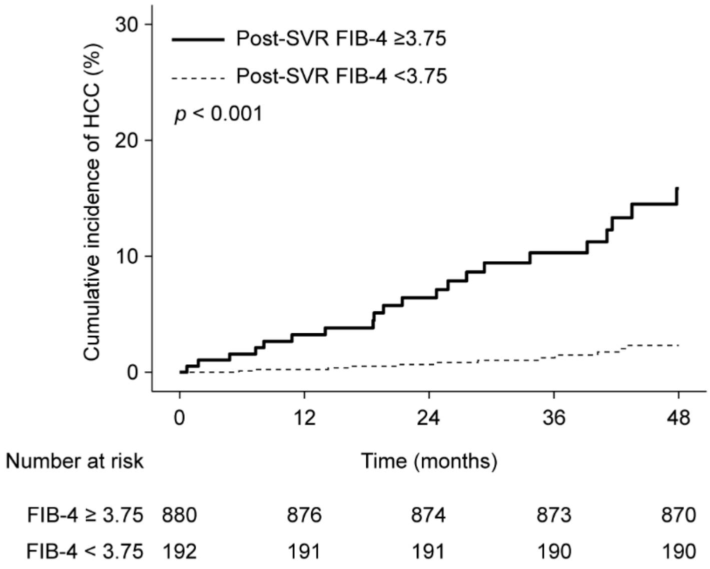

Figure S3. Cumulative probabilities of HCC between patients with FIB-4 ≥ 3.75 and those with FIB-4 < 3.75.
